# The hantaviruses of Europe: from the bedside to the bench.

**DOI:** 10.3201/eid0302.970218

**Published:** 1997

**Authors:** J Clement, P Heyman, P McKenna, P Colson, T Avsic-Zupanc

**Affiliations:** Queen Astrid Military Hospital, Brussels, Belgium.

## Abstract

In Europe, hantavirus disease can hardly be called an emerging zoonosis; it is rather a rediscovered disease. Since 1934 an epidemic condition with primarily renal involvement has been described in Sweden. Nowadays, hundreds to thousands of cases per year are registered in Fennoscandia, fluctuating with the numbers of the specific Arvicoline-rodent reservoir, the red bank vole, which carries the main European serotype, Puumala (PUU). In the early 1980s, the rat-transmitted serotype, Seoul (SEO), caused laboratory outbreaks throughout Europe, and recent reports also suggest sporadic, wild rat-spread hantavirus disease. In the Balkans, at least four serotypes are present simultaneously: PUU, SEO, the "Korean" prototype Hantaan (HTN) or HTN-like types, and Dobrava, the latter causing a mortality rate of up to 20%. Moreover, recent genotyping studies have disclosed several PUU-like genotypes spread in Europe and/or Russia by other genera of the Arvicoline-rodent subfamily: Tula, Tobetsu, Khabarovsk, and Topografov. Their importance for human pathogenicity is still unclear, but serologic cross-reactions with PUU antigen might have caused their misdiagnosis as PUU-infections in the past.


The articles in this section were originally presented at the 1st International Conference on Emerging Zoonoses, Jerusalem, Israel, November 24-28, 1996. The conference was cosponsored by the Centers for Disease Control and Prevention and the Israel Center for Disease Control.

### The Hantaviruses of Europe: from the Bedside to the Bench

In Europe, hantavirus disease can hardly be called an emerging zoonosis; it is rather a rediscovered disease. Since 1934 an epidemic condition with primarily renal involvement has been described in Sweden. Nowadays, hundreds to thousands of cases per year are registered in Fennoscandia, fluctuating with the numbers of the specific *Arvicoline*-rodent reservoir, the red bank vole, which carries the main European serotype, Puumala (PUU). In the early 1980s, the rat-transmitted serotype, Seoul (SEO), caused laboratory outbreaks throughout Europe, and recent reports also suggest sporadic, wild rat-spread hantavirus disease. In the Balkans, at least four serotypes are present simultaneously: PUU, SEO, the "Korean" prototype Hantaan (HTN) or HTN-like types, and Dobrava, the latter causing a mortality rate of up to 20%. Moreover, recent genotyping studies have disclosed several PUU-like genotypes spread in Europe and/or Russia by other genera of the *Arvicoline*-rodent subfamily: Tula, Tobetsu, Khabarovsk, and Topografov. Their importance for human pathogenicity is still unclear, but serologic cross-reactions with PUU antigen might have caused their misdiagnosis as PUU-infections in the past.

Hantaviruses are often heralded as new or at least as emerging pathogens, particularly in the New World. However, even on the American continent, application of the newest genotyping techniques (the bench) comparing RNA sequences found in both human cases and rodent reservoirs shows a long-standing coevolution of each newly discovered hantavirus serotype in its specific rodent host; this coevolution results in remarkable genetic stability across time and in a certain genetic differentiation in geographic spread. Hantaviruses inducing hantavirus pulmonary syndrome emerge not through genetic reassortment or a recent mutation, but through increased exposure to infected rodents and their excreta. While the same lines of evidence also apply to the European situation, the problem there is totally different, from a historical point of view.

### History

In most European countries, hantavirus disease has long been known by various (mostly geographic) names, which suggests a long-standing clinical presence (the bedside). An epidemic of "trench nephritis" during World War I may in fact have been hantavirus induced. Thousands of cases of this illness, considered an entirely new renal disease, were noted on both sides of the front (Kriegsnephritis or néphrite de guerre). Already in 1934, the typical mild renal form of hantavirus disease had been described in Sweden (1); it was then described in all other Scandinavian countries, where the disease was appropriately called nephropathia epidemica from 1945 on.

During World War II, more than 10,000 cases of a rodent-borne leptospirosislike disease were noted during the 1942 German campaign in Finnish Lapland (2). Because the snow melted, great numbers of lemmings and field mice invaded the German bunkers. Examinations in Munich and Berlin of these rodents, air-lifted from the war theater, offered no clue. Confronted with some distinctive clinical symptoms (e.g., acute myopia and localized edema) and with repeatedly negative findings for leptospirosis in his patients, a researcher concluded it was a new field-like fever disease (2).

### "Old" Hantaviruses in the Old World

The Puumala (PUU) serotype, carried by the red bank vole (*Clethrionomys glareolus*), remains the most important Western and Central European serotype, with at least 1,000 serologically confirmed nephropathia epidemica cases per year in Finland and hundreds per year in Sweden (3). The number of documented cases in other European countries was more than 1,000 in the former Yugoslavia (3), 531 in France by the end of 1994 (4), approximately 250 in Belgium by the end of 1996 (J. Clement, unpub. obs.), some 200 in Germany by the end of 1995 (5), 138 in Greece by the end of 1993 (6), and 39 in the Netherlands by the end of 1994 (7).

IgG seroprevalence rates reported from some of these countries, measured mostly by immunofluorescence assay (IFA) and/or enzyme-linked immunosorbent assay (ELISA) both for PUU and HTN, were 6% for Finland (8), 8% for Sweden (9), 1.7% for Germany (10), 0.9% for the Netherlands (11), 1.6% for Belgium (12), <1% for France (4), 0.3% for Spain (13) and 4.0% for Greece (6). Most of the PUU infections were subclinical. A study comparing nephropathia epidemica incidence (recorded over 14 years) with IgG IFA PUU-antibody prevalence in an area of Sweden with high rates of endemic disease found that the antibody prevalence rate for men and women in the oldest age groups (>60 years) was 14 to 20 times higher, respectively, than the accumulated life-risk of being hospitalized with nephropathia epidemica (14). Thus, hospital admissions for the disease are only the tip of the iceberg.

In nephropathia epidemica, acute renal failure serum creatinine values peak above 4.5 mg (>400 mol/L) in only one-third of the cases (15). Early thrombocytopenia, however, is present in 75% of the cases. Eye symptoms, and particularly acute myopia, are rare (25%) but distinctive first symptoms of PUU infections. Mild transient hepatitis is frequent (40%), but icterus is rare (7%) (15). Noncardiogenic pulmonary edema, the hallmark of Sin Nombre (or Sin Nombre-like)-induced hantavirus disease in the New World, has been described in a milder, nonlethal form (acute lung injury) in some rare European PUU (and Seoul [SEO] virus) cases (16,17).

In the former Soviet Union, hantavirus disease has been recognized since 1934 and officially registered since 1978. Seroprevalence studies carried out by IFA or direct blocking radioimmunoassay involving 115,765 persons resulted in an overall seropositivity rate of 3.3%, ranging from 3.5% in the European part to 0.9% in the Far Eastern part (18). A total of 68,612 cases were registered between 1988 and 1992 (65,906 from the European part and 2,706 from the Far Eastern part), with morbidity rates of 1.2 (1982) to 8.0 (1985) per 100,000 inhabitants. The peak year was 1985 with 11,413 registered cases (19). In the European part of Russia, most cases were due to milder infection with PUU-related viruses, with mortality rates of 1% to 2%; whereas in Far East Russia, more severe HTN-like cases also occurred (19).

### The Wild Rat: Omnipresent, but Often Overlooked

In Europe, the parallel spread of PUU and HTN (or HTN-like) viruses has been noted in such countries as Belgium and the Netherlands (11,20), Germany (10), and European Russia (21). A partial explanation could be that the HTN-like infection is due to serologic cross-reaction with SEO. The wild rat is the only hantavirus reservoir with a worldwide distribution (22), including Europe, and SEO infections are probably underestimated. The first documented hantavirus disease in Portugal was an HTN-like infection with acute renal failure and icterus (23). In Portugal *Clethrionomys glareolus* is not prevalent, but hantavirus-seropositive wild rats have been documented (24). Sixteen cases of acute disease, mostly with acute renal failure and reacting almost exclusively in IFA against a SEO strain (R22VP30), have been described in North Ireland (25), another country where *C. glareolus* is not prevalent, but the most important hantavirus vector seems to be the wild rat (26). In France, three SEO-induced cases of acute renal failure have been reported south of the PUU-endemic region, from rural areas where the rat is an agricultural pest (4). Moreover, 14 SEO-like cases were detected between December 1991 and February 1992 in the Tula region (300 km south of Moscow) and confirmed by plaque reduction neutralization tests and positive virus isolation in three of the cases (27); these cases are awaiting further confirmation.

An often overlooked fact is that hantavirus has been transmitted from laboratory rats to animal keepers first in Belgium (1979) and later in France, in the United Kingdom, and in the Netherlands (28). In the earlier days, these rat-transmitted infections were described as HTN-like by IFA or

ELISA because of cross-reactions with the prototype screening antigen HTN 76-118, but they were later confirmed as SEO-like by ELISA and blocking ELISA (20). Because of the now established close relationship between each hantavirus serotype and its rodent vector, these earlier laboratory infections can all be regarded as SEO-induced.

### The Balkans: A Complicated Situation

In the Balkans, and particularly the former Yugoslavia, outbreaks of hantavirus disease have been recorded since the early 1950s, often with a death rate of 5% to 10% or even higher (29). The elevated rates of illness and death in early reports suggested the spread of one (or several) hantaviral strains, in addition to the mild PUU serotype prevalent in the rest of Europe. These HTN-like viruses were later called Plitvice and Fojnica. In 1987, an HTN-like virus (Porogia) was isolated from the urine of a Greek soldier who became ill after a military exercise near the border in northern Greece and had both acute renal failure and severe pulmonary edema. Extensive cross-reactivity with HTN 76-118 was demonstrated by IFA with a panel of Mabs and in plaque reduction neutralization tests (30). No polymerase chain reaction (PCR) genotyping was available at that time. However, in Slovenia in 1994, a hantavirus was isolated that was indistinguishable from the prototype Korean strain HTN 76-118 by PCR genotyping and other serologic techniques (31). Moreover, a hantavirus (close but not identical to HTN 76-118) that caused a mortality rate of up to 20% was first described as a human isolate (Belgrade) in Serbia (32) and confirmed as a new hantavirus serotype called Dobrava (DOB) after isolation from its rodent vector, an *Apodemus flavicollis* (yellow-necked field mouse) captured in Dobrava, Slovenia (33).

The first genetic evidence for the association between DOB and severe hantavirus disease was demonstrated by nested reverse transcriptase-PCR on RNA extracted from whole blood of a Greek and an Albanian patient (34). During the recent conflict in Bosnia, more than 300 patients, most of them soldiers exposed in the field, were hospitalized in the Tuzla region (northeast Bosnia) with acute hantavirus disease due either to PUU or to DOB, as first documented by IgG and IgM ELISA (35) and later confirmed by focus reduction neutralization tests (36). These findings suggest that at least three distinct serotypes (PUU, DOB, and maybe also HTN) are endemic throughout the Balkans and that easily accessible serologic tests are needed to permit a differential diagnosis, given the totally different prognosis for each infection. Moreover, preliminary evidence implicated a fourth serotype, i.e., SEO, spread by wild rats (*Rattus norvegicus* or *Rattus rattus).* Severe hantavirus disease, apparently due to SEO, was documented in 1992 in a Canadian soldier (37) and later in 1996 in a British soldier (17), both stationed in Bosnia. These two patients are clinically interesting, in that the former had a clear exposure to wild rats inside an infested building, whereas the latter had acute renal failure and hemodynamically documented acute lung injury, a complication hitherto undocumented in SEO cases (38). The exact serotype involved in both these cases needs to be defined, however, by confirmatory tests such as plaque reduction neutralization tests or (if technically possible) by PCR genotyping.

That so many of these Balkan cases are in persons on active military duty should come as no surprise. Exposure to rodents has been confirmed as the most important risk factor for developing hantavirus diseases and seems an unavoidable aspect in the life of the soldier at war. Even exercises imitating war conditions can put the soldier at risk: the most important cluster of hantavirus disease in Americans abroad was reported in U.S. soldiers exercising in January 1990 in southern Germany and camping under tent in a mice-infested area. Within 2 weeks, 24 acute PUU infections were documented, and 14 soldiers had to be hospitalized with varying degrees of acute renal failure (no deaths), whereas no outbreak occurred in the civilian population of the surrounding area (5).

### "New" Hantaviruses: Tula (TUL), Tobetsu (TOB), Khabarovsk (KBR), and Topografov (TOP)

Apart from the long-standing clinical experience with hantavirus strains in Europe and Asia, an explosive growth in the number of newly discovered lineages or genotypes has further complicated matters. All these new genotypes appear more or less related to PUU (Figure, Table). TUL virus was first detected by RT-PCR in European common voles (*Microtus arvalis* and *Microtus rossiaemeridionalis*) captured in the Tula region (39). TUL virus was later also detected in voles from Moravia, the Czech Republic, and Slovakia (Figure). The three viruses most closely related to TUL have been detected on the American continent: Prospect Hill, isolated in 1982 from *Microtus pennsylvaticus* (meadow vole); Isla Vista virus, recently detected in *Microtus californicus* (Californian meadow vole) (40); and Bloodland Lake virus, detected in *Microtus ochrogaster* (prairie vole) (Hjelle et al., unpub. data). TUL was isolated from the lungs of infected *M. arvalis* and showed in cross-focus reduction neutralization tests and cross-hemagglutination inhibitor tests at least eightfold higher homologous to heterologous titers when compared with PUU, PH, KBR, and HTN (41). None of these *Microtus*-derived hantaviruses is a known pathogen in humans, in contrast to the PUU viruses, which are spread by another genus of the same rodent subfamily *Arvicolinae* (Table, Figure). However, serum of a blood donor living in Moravia, the Czech Republic, possessed a focus reduction neutralization test titer to TUL at least 16-fold higher than to PUU or other hantaviruses, thereby giving the first solid evidence that these viruses carried by *Microtus* rodents can infect humans (41). As in other hantavirus serotypes, antibody response to the TUL N-antigen appeared highly reactive and cross-reactive. Thus, part of the so-called PUU infections in European (and particularly in Central European and Russian) patients may have been due to related TUL viruses. Under that hypothesis, it would be very remarkable that the even more closely related North American viruses (Prospect Hill, Isla Vista, and Bloodland Lake) would appear to be apathogenic to humans. Already in the early 1930s, Tula fever was one of the many regional synonyms used in Russia for describing epidemics of a feverish condition, which later appeared to be a hantavirus infection.

TOB was the name preliminarily given to a PUU-like virus detected in *Clethrionomys rufocanus* (grey-sided vole) captured in Hokkaido, an island in the north of Japan (44). Its putative rodent reservoir, *Cl. rufocanus*, has a very broad geographic range, extending almost over the whole of Eurasia: in the north from northern Scandinavia to Kamtchatka, and in the south from the Urals to Manchuria and down to Korea. Moreover, PUU-like human infections have been noted in Korea (18) (H.W. Lee, pers. comm.).

KBR (not in the Figure), a hantavirus close to PUU, was recently isolated from a *Microtus fortis* (reed vole) captured in Far East Russia (46). Although no pathogenicity for humans has been documented so far, this new agent could partially explain PUU-like infections described for many years in this region and in China, together with the findings of PUU-positive *M. arvalis* rodents (19). KBR is more closely related to PUU than to Prospect Hill, which may reflect that both KBR and PUU are viruses from the Old World, whereas Prospect Hill has been documented so far only in North America. The first reports in Russia that describe a hantaviruslike disease are found in the 1913 archives of a hospital in Vladivostok, Siberia.

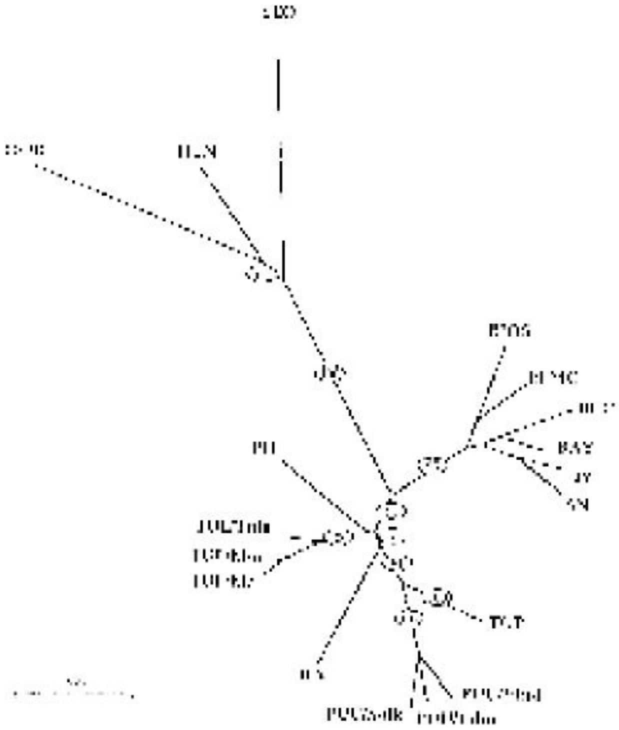

Figure: Dendrogram of Old World hantaviruses (upper, left, and lower part of the tree) vs. New World hantaviruses (right part of the tree). Reproduced with permission (42). Branch lengths are proportional to genetic distances. The bootstrap support percentages of particular branching points calculated from 500 replicates are given in ovals. HTN = Hantaan virus, strain 76-118; SEO = Seoul virus, strain SR-11; DOB = Dobrava virus, PH = Prospect Hill virus; TUL = Tula virus, strains Tula/76Ma/87, Moravia/5286Ma/94 and Malacky/Ma32/94; ILV = Isla Vista virus, strain MC-SB-1; TOP = Topografov virus; PUU = Puumala virus, strains Sotkamo, Vindeln/83-L20 and Udmurtia/458g/88; RIOS = Rio Segundo virus, strain RMx-Costa-1; ELMC = El Moro Canyon virus, strain RM-97; BAY = Bayou virus, strain Louisiana; BCC = Black Creek Canal virus; SN = Sin Nombre virus, strain H10; NY = New York virus, strain RI-1; KBR = Khabarovsk virus, another PUU-like virus, is not depicted here. PH & ILV (and BLL, not depicted here) are the only New World genotypes with PUU-like characteristics, hence their position close to the TUL-clade.

Table: Classification of old and new PUU-like genotypes, according to respective rodent host and geographic spread.

| Rodent reservoir (*Arvicolinae* Subfamily) | Hantavirus (geographic spread) | Reference |
|---|---|---|
| *Clethrionomys* | | |
| *glareolus* | Puumala (Europe) | (43) |
| *rufocanus* | Tobetsu (Japan) | (44) |
| *Lemmus* | | |
| *sibiricus* | Topografov (Russia) | (42) |
| *Microtus* | | |
| *pennsylvaticus* | Prospect Hill (N. America) | (45) |
| *californicus* | Isla Vista (N. America) | (40) |
| *ochrogaster* | Bloodland Lake (N. America) | Hjelle et al., unpublished data |
| *arvalis* | Tula (Eurasia) | (39) |
| *rossiaemeridionalis* | Tula (Eurasia) | (39) |
| *fortis* | Khabarovsk (Eurasia) | (46) |

TOP is another PUU-like genotype, detected in the lemming (*Lemmus sibiricus*) (42). Lemmings, the most important small mammals in the Arctic tundra regions, are also present in Alaska and Canada. Together with the wild rat, the lemming is the only rodent reservoir harboring a newly recognized hantavirus genotype, and living in both the Old and the New World. Of the newer European viruses, TOP is the most closely related to PUU (Figure). No human pathogenicity has been recognized. However, lemming fever has traditionally been reported by Nordic inhabitants, particularly during lemming years (42); this link was made already in 1942 (another lemming year) by Stuhlfauth (2) when describing an epidemic in German troops plagued by lemmings.

With the current explosive growth of knowledge concerning hantaviruses, a tendency is emerging to globalize at least some strains and symptoms: 1) The distribution of various new and old strains is giving an ever more confused picture of infection in Eurasia, with HTN-like strains (HTN and DOB) in the West, and PUU-like strains (TUL, TOB, KBR, and TOP) in the East. Moreover, Bloodland Lake and Isla Vista have joined the prototype North American isolate Prospect Hill as PUU-like strains in the Americas. 2) The wild rat SEO-strain remains probably the most underestimated (pathogenic) hantavirus strain worldwide, despite recent reports of SEO-like infections throughout Europe and the Americas. 3) The clinical symptoms tend also to grow to each other on the global scene: whereas in the Americas, non-Sin Nombre virus cases of hantavirus pulmonary syndrome, i.e., induced by Black Creek Canal virus and/or Bayou virus, have renal as well as lung involvement, and whereas even mild cases have recently been described, we also find now, albeit rarely, evidence of lung involvement under the form of acute lung injury in documented PUU and SEO cases in Europe. 4) Careful reading of the earlier literature, often containing astute clinical or epidemiologic descriptions of viral hemorrhagic fevers, can still teach us many lessons, both for the bedside and for the bench.

### Acknowledgment

This work was made possible by a grant JSM R&D 96/01 of the Belgian Army.

**Jan Clement,* Paul Heyman,* Paula McKenna,* Paul Colson,† and Tatjana Avsic-Zupanc‡**
*Queen Astrid Military Hospital, Brussels, Belgium; †Centre de Santé des Fagnes, Chimay, Belgium; and ‡University of Ljubljana, Ljubljana, Slovenia

### References

1. Myhrman G. A renal disease with particular symptoms. Nordisk Medicinsk Tidskrift 1934;7:793-4.
2. Stuhlfauth K. Bericht über ein neues schlammfieberähnliches Krankheitsbild bei Deutscher Truppen in Lappland. Deutsche medizinische Wochenschrift 1943;439:474-7.
3. Mustonen J, Vaheri A, Clement J. Congress report: Third International Conference on Haemorrhagic Fever with Renal Syndrome (HFRS) and Hantaviruses. Nephrol Dial Transplant: 1996;11:730-3.
4. Le Guenno B, Coudrier D. Epidemiology of hantavirus infections in France (1977-1995). Proceedings of the Third International Conference on HFRS and Hantaviruses 1994 May 31-June 3; Helsinki, Finland. Helsinki: Haartman Institute, University of Helsinki, 1994;11.
5. Clement J, Underwood P, Ward D, Pilaski J, LeDuc J. Hantavirus outbreak during military manoeuvres in Germany. Lancet 1996;347:336.
6. Papadimitriou MG, Antoniadis A. Hantavirus nephropathy in Greece. Lancet 1994;343:1038.

7. Gerding M, Groen J, Jordans JGM, Osterhaus ADME. Hantavirus nephropathy in the Netherlands: clinical, histopathological and epidemiological findings. Neth J Med 1995;47:106-12.
8. Vapalahti O, Vaheri A, Henttonen H. Eurosurveillance. Commission of the European Communities, Brussels, Belgium; 1995 Sept. European Communicable Disease Bulletin, No.0:3-4.
9. Niklasson B, LeDuc J. Epidemiology of nephropathia epidemica in Sweden. J Inf Dis 1987;155:269-76.
10. Zöller L, Faulde M, Meisel H, Ruh B, Kimmig P, Schelling U, et al. Seroprevalence of hantavirus antibodies in Germany as determined by a new recombinant enzyme immunoassay. Eur J Clin Microbiol Infect Dis 1995;14:305-13.
11. Groen J, Gerding MN, Jordans JG, Clement JP, Nieuwenhuijs JH, Osterhaus AD. Hantavirus infections in The Netherlands: epidemiology and disease. Epidemiol Infect 1995;114:373-83.
12. Clement J, van der Groen G. Acute Hantavirus nephropathy in Belgium: preliminary results of a seroepidemiological study. In: Amerio A, Coratelli B, editors. Acute Renal Failure. Advances in experimental medicine and biology. New York: Plenum Press, 1987:251-63.
13. Rodriguez JA, Vaque J. Hantavirus disease: an emerging infection [editorial]. Enferm Infecc Microbiol Clin 1994;12:477-9.
14. Niklasson B, Leduc JW, Nystrom K, Nyman L. Nephropathia epidemica: incidence of clinical cases and antibody prevalence in an endemic area of Sweden. Epidemiol Infect 1987;99:559-62.
15. Colson P, Damoiseaux P, Brisbois J, Duvvier E, Levecque P., Roger JM, et al. Hantavirose dans l'Entre-Sambre-et-Meuse. Acta Clin Belg 1995;50:197-206.
16. Clement J, Colson P, McKenna P. Hantavirus pulmonary syndrome in New England and Europe. N Engl J Med 1994;331:545-6.
17. Stuart LM, Rice PS, Lloyd G, Beale RJ. A soldier in respiratory distress. Lancet 1996;347:30.
18. World Health Organization Working Group on the development of a rapid diagnostic method and vaccine for hemorrhagic fever with renal syndrome. Seoul, Republic of Korea, 1991 Sep 26-28; WPR/OCD/CDS(O)/1/91/IB3.
19. Haemorrhagic fever with renal syndrome. Wkly Epidemiol Rec 1993;68:189-91.
20. Groen J, Jordans H, Clement J, Rooijakkers E, Uytdehaag F, Dalrymple J, et al. Identification of hantavirus serotypes by testing of post-infection sera in immunofluorescence and enzyme-linked immunosorbent assays. J Med Virol 1991;33:26-32.
21. Alexeyev O, Elgh F, Zhestkov A, Wadell G, Juto P. Hantaan and Puumala virus antibodies in blood donors in Samara, an HFRS-endemic region in European Russia. Lancet 1996;347:1483.
22. LeDuc JW, Smith GA, Childs JE, Pinheiro FP, Maiztegui JI, Niklasson B, et al. Global survey of antibody to hantaan related viruses among peridomestic rodents. Bull World Health Organ 1986;64:139-44.
23. Monteiro J, Mesquita M, Alves MJ, Filipi AR. Febre Hemorragica com Sindroma Renal - Primeiro caso clinico diagnosticado em Portugal. Separata da Revista Portuguesa de Doenas Infecciosas. 1993;16:209-14.
24. Filipe AR, Andrade HR, Sommer AI, Traavik T. Hantaviral antigens and antibodies in wild rodents in Portugal. Acta Virol 1991;35:287-91.
25. McKenna P, Clement J, Matthys P, Coyle PV, McCaughey C. Serological evidence of hantavirus disease in Northern Ireland. J Med Virol 1994;43:33-8.
26. McCaughey C, Montgomery WI, Twomey N, Addley M, O'Neill HJ, Coyle PV. Evidence of hantavirus in wild rodents in Northern Ireland. Epidemiol Infect 1996;117:361-5.
27. Dzagurova T, Myasnikov Y, Dekonenko A, Tkachenko E. Seoul-type hantavirus isolated from HFRS patient in European Russia. Proceedings of the Third International Conference on HFRS and Hantaviruses; 1994 May 1-June 3; Helsinki, Finland. Helsinki: Haartman Institute, University of Helsinki, 1994; 69..
28. McKenna P, van der Groen G, Hoofd G, Beelaert G, Leirs H, Verhagen R. Eradication of hantavirus infection among laboratory rats by application of caesarian section and a foster mother technique. J Infect 1992;25:181-90.
29. Heneberg D, Vuksic L, Morelj M, Lepes I, Djordjevic Z, Mikes M, et al. Epidemic of hemorrhagic fever in certain workplaces in Fruska Gora. Zbornik radova VMA 1962:236-71.
30. Antoniadis A, Greekas D, Rossi CA, LeDuc JW. Isolation of a hantavirus from a severely ill patient with hemorrhagic fever with renal syndrome in Greece. J Infect Dis 1987;156:1010-3.
31. Avsic-Zupanc T, Poljak M, Furlan P, Kaps R, Shu Yuan Xiao, LeDuc JW. Isolation of a strain of a hantaan virus from a fatal case of hemorrhagic fever with renal syndrome in Slovenia. Am J Trop Med Hyg 1994;51:393-400.
32. Gligic A, Dimkovic N, Xiao SY, Buckle GJ, Jovanovic D, Velimirovic D, et al. Belgrade virus: a new hantavirus causing severe hemorrhagic fever with renal syndrome in Yugoslavia. J Infect Dis 1992;166:113-20.
33. Avsic-Zupanc T, Xiao S-Y, Stojanovic R, Gligic A, van der Groen G, LeDuc JW. Characterisation of Dobrava virus: a hantavirus from Slovenia, Yugoslavia. J Med Virol 1992;38:132-7.
34. Antoniadis A, Stylianakis A, Papa A, Alexiou-Daniel S, Lampropoulos A, Nichol ST, et al. Direct genetic detection of Dobrava virus in Greek and Albanian patients with hemorrhagic fever with renal syndrome. J Infect Dis 1996;174:407-10
35. Hukic M, Kurt A, Torstensson S, Lundkvist A, Wiger D, Niklasson B. Haemorrhagic fever with renal syndrome in north-east Bosnia. Lancet 1996;347:56-7.
36. Lundkvist A, Hukic M, Hörling J, Gilljam M, Nichol S, Niklasson B. Puumala and Dobrava viruses cause haemorrhagic fever with renal syndrome (HFRS) in Bosnia-Herzegovina: evidence of highly cross-neutralizing antibody responses in early patient sera. J Med Virol. In press.
37. Clement J, Mc Kenna P, Avsic Zupanc T, Skinner CR. Rat-transmitted hantavirus disease in Sarajevo. Lancet 1994;344:131.
38. Clement J, Heyman P, Colson P, Groeneveld PH. Spread of hantavirus infections in Europe. Lancet 1996;347:771.

39. Plyusnin A, Vapalahti O, Lankinen H, Lehvaslaiho H, Apekina N, Myasnikov Y, et al. Tula virus : a newly detected hantavirus carried by European common voles. J Virol 1994;68:7833-9.
40. Song W, Torrez Martinez N, Irwin W, Harrison FJ, Davis R, Ascher M, et al. Isla Vista virus: a genetically novel hantavirus of the California vole *Microtus californicus*. J Gen Virol 1995;76:3195-9.
41. Vapalahti O, Lundkvist A, Kukkonen S, Cheng Y, Giljam M, Kaverna M. Isolation and characterisation of Tula virus, a distinct serotype in the genus Hantavirus, family Bunyaviridae. J Gen Virol. In press.
42. Plyusnin A, Vapalahti O, Lundkvist A, Henttonen H, Vaheri A. Newly recognized hantavirus in Siberian lemmings. Lancet 1996;347:1835.
43. Brummer Korvenkontio M, Vaher A, Hovi T, von Bonsdoff C, Vuorimies J, Manni T, et al. Nephropathia epidemica: detection of antigen in bank voles and serologic diagnosis of human infection. J Infect Dis 1980;141:131-4.
44. Kariwa H, Yoshizumi S, Arikawa J, Yoshimatsu K, Takahashi K, Takashima I, et al. Evidence for the existence of Puumula-related virus among *Clethrionomys rufocanus* in Hokkaido, Japan. Am J Trop Med Hyg 1995;53:222-7.
45. Lee PW, Amyz HL, Gajdusek DC, Yanagihara RT, Goldgaber D, Gibbs CJ. New haemorrhagic fever with renal syndrome-related virus in indigenous wild rodents in United States. Lancet 1982;2:1405.
46. Hörling J, Chizhikov V, Lundkvist A, Jonsson M, Ivanov L, Dekonenko A, et al. Khabarovsk virus: a phylogenetically and serologically distinct hantavirus isolated from *Microtus fortis* trapped in far-east Russia. J Gen Virol 1996;77:687-94.
